# Absence of Fibrosis Influences Prognosis of Cardiac Resynchronization in Nonischemic Cardiomyopathy: Systematic Review and Meta-Analysis

**DOI:** 10.1016/j.jacadv.2026.103295

**Published:** 2026-10-01

**Authors:** Jesús Sánchez-Vega, Ignasi Anguera, Francisco Leyva, Otto A. Smiseth, Jens-Uwe Voigt, Juan Fernandez-Armenta, Markus Linhart, Antonio Berruezo, Shunmuga Sundaram Ponnusamy, Arnaud Bisson, Giulio Conte, Francesco Maffessanti, Alejandro Ruiz-Majoral, Marta Gonzalez-Lopera, Eduard Claver-Garrido, Laia Lorenzo-Esteller, Fernando de Frutos, Gemma Mateus-Porta, Jesus Rodriguez-Silva, Josep Comin-Colet, Andrea Di Marco

**Affiliations:** a Department of Cardiology, Bellvitge University Hospital, Hospitalet de Llobregat, Barcelona, Spain; b Bioheart Group, Cardiovascular, Respiratory and Systemic Diseases and Cellular Aging Program, Institut d’Investigació Biomèdica de Bellvitge (IDIBELL), L’Hospitalet de Llobregat, Barcelona, Spain; c University Hospitals Birmingham Queen Elizabeth, Birmingham, United Kingdom; d Division of Cardiovascular and Pulmonary Diseases, Institute for Surgical Research, Oslo University Hospital, Rikshospitalet, Oslo, Norway; e Faculty of Medicine, University of Oslo, Oslo, Norway; f Department of Cardiovascular Diseases, University Hospitals Leuven, Leuven, Belgium; g Department of Cardiology, Puerta del Mar University Hospital, Cádiz, Spain; h Cardiology Department, University Hospital Doctor Josep Trueta, Girona, Spain; i Quironsalud Group, Teknon Medical Center, Arrhythmia Department, Heart Institute, Barcelona, Spain; j Department of Cardiology, Velammal Medical College Hospital and Research Institute, Madurai, India; k Service de Cardiologie, Centre Hospitalier Régional Universitaire et Université de Tours, Tours, France; l Cardiocentro Ticino Institute, Ente Ospedaliero Cantonale, Lugano, Switzerland; m Maria Cecilia Hospital, GVM Care & Research, Cotignola, Italy; n Innovation, Research and Universities Department, Gerència Territorial Metropolitana Sud, Institut Català de la Salut, Spain; o Department of Clinical Sciences, School of Medicine, University of Barcelona, Barcelona, Spain; p Centro de Investigación Biomédica en Red de Enfermedades Cardiovasculares, CIBER-CV, Madrid, Spain

**Keywords:** cardiac magnetic resonance, myocardial fibrosis, ventricular arrhythmia, sudden cardiac death, implantable cardioverter-defibrillator, reverse remodeling

## Abstract

**Background:**

In patients with nonischemic cardiomyopathy (NICM) undergoing cardiac resynchronization therapy (CRT), there are no clear recommendations on selecting CRT with defibrillator (CRT-D) vs pacemaker (CRT-P). Patients with high probability of response to CRT and low arrhythmic risk may be ideal candidates to CRT-P.

**Objectives:**

The objectives of the study was to evaluate if the absence of late gadolinium enhancement (LGE) is associated with CRT response and ventricular arrhythmia (VA) in NICM treated with CRT.

**Methods:**

A systematic review and meta-analysis was conducted. Search of PubMed, Web of Science, and Scopus identified observational studies of NICM patients who underwent cardiac magnetic resonance before CRT implantation. Studies assessing echocardiographic response to CRT or VA stratified by LGE status were included. The VA endpoint comprised sustained ventricular tachycardia, appropriate implantable cardioverter-defibrillator therapy, resuscitated cardiac arrest, and sudden death.

**Results:**

Ten studies were included: 9 (n = 831) for CRT response and 6 (745 patients) for arrhythmic risk. CRT response was significantly higher in patients without LGE than in patients with LGE (78.7% vs 50.7%; *P* < 0.001). The cumulative incidence of VA was extremely low in LGE cases, significantly lower than that of LGE+ ones (0.8% vs 21.1%, *P* < 0.001). The absence of LGE was strongly associated with higher CRT response (OR: 3.52; 95% CI: 2.54-4.88; *P* < 0.001) and much lower arrhythmic risk (OR: 0.06; 95% CI: 0.02-0.15; *P* < 0.001).

**Conclusions:**

In NICM patients undergoing CRT, the absence of LGE is strongly associated with greater echocardiographic response and very low arrhythmic risk. In this subgroup the incremental benefit of CRT with defibrillator over CRT-P may be limited.

In patients with nonischemic cardiomyopathy (NICM) who are candidates to cardiac resynchronization therapy (CRT) as primary prevention, CRT with defibrillator (CRT-D) has not consistently demonstrated superior survival over CRT with pacemaker (CRT-P).^1^ Current clinical guidelines do not specify which subgroup of CRT candidates could be adequately managed with CRT-P alone; actually, according to guidelines, the majority of candidates to CRT with left ventricular ejection fraction (LVEF) ≤35% also has an indication for primary prevention implantable cardioverter-defibrillator (ICD) and, by consequence, should receive a CRT-D.^2^^,^^3^

However, patients with a low risk of ventricular arrhythmias (VAs) and high probability of echocardiographic response to CRT may represent ideal candidates to CRT-P.

In NICM, the absence of myocardial scar, as detected by cardiac magnetic resonance (CMR) with late gadolinium enhancement (LGE), is associated with low risk of VA and sudden death^4^, ^5^, ^6^, ^7^ and with higher probability of reverse remodeling in response to heart failure pharmacotherapy.^8^ If these results were confirmed in patients with CRT, those without LGE could be potential candidates to CRT-P.

Several observational studies have analyzed the prognostic impact of LGE in NICM patients with CRT, evaluating its association with VA and/or echocardiographic response.^9^, ^10^, ^11^ However, the majority of these works were single-center projects including relatively small cohorts.

Therefore, we considered relevant to perform this meta-analysis to provide a comprehensive and detailed evaluation of the association between the absence of LGE and both VA and echocardiographic response to CRT in patients with NICM treated with CRT.

## Methods

This study involved the secondary use of existing, publicly available, and deidentified data. According to the European General Data Protection Regulation, such studies do not require formal ethics approval.

This systematic review and meta-analysis was conducted in accordance with the principles of the PRISMA (Preferred Reporting Items for Systematic Reviews and Meta-Analyses) 2020 statement^12^ (checklist in Supplemental Table 1).

### Search strategy

A thorough bibliographic search was conducted by 2 investigators (A.D.M. and J.S.V.) to identify studies meeting the inclusion criteria in the PubMed (MEDLINE), Web of Science, and Scopus databases up to November 2024.

The search strategy combined controlled vocabulary and free-text terms related to CRT, CMR, and LGE. The complete search strategy for each database is provided in the Supplemental Table 2. No language restrictions were applied. To ensure saturation, the reference lists of included reports and relevant reviews were also scanned.

### Inclusion criteria

The research question was formulated according to the Population, Exposure, Comparison, Outcome, and Study design framework, with each component defined as follows.

We included observational studies enrolling adult patients (age ≥18 years) with NICM who underwent CRT implant (either CRT-P or CRT-D), including both traditional resynchronization with a left ventricular lead in a coronary vein and left bundle branch area pacing. For analyses of arrhythmic outcomes, eligibility was restricted to cohorts of primary prevention patients. The exposure of interest was CMR with gadolinium administration before CRT implantation. Comparison groups were defined according to the presence or absence of LGE, considering any nonischemic LGE pattern.

### Outcomes

The outcomes of interest were: 1) echocardiographic response to CRT (according to the definition used in each study); and 2) VA, including sudden cardiac death, resuscitated cardiac arrest, ventricular fibrillation, sustained ventricular tachycardia, and appropriate ICD therapies.

### Study selection and data collection process

Observational cohort studies, both prospective and retrospective, were eligible for inclusion. Studies enrolling patients with NICM were considered. In studies that also included patients with ischemic cardiomyopathy, only data from the NICM subgroup were analyzed.

Two investigators (A.D.M. and J.S.V.) independently screened titles and abstracts. Full texts of potentially relevant articles were subsequently assessed for eligibility. Discrepancies were resolved by consensus. Data extracted included study design, population characteristics, interventions, comparators, sample size, and outcomes. Reasons for study exclusion were documented.

Raw data on the number of patients with and without echocardiographic response to CRT or with and without arrhythmic events, stratified by the presence or absence of LGE, were extracted from the original reports or obtained by contacting the corresponding authors.

Additional variables collected included study design, study population size, LGE assessment method, endpoint definitions, NICM definition, follow-up duration, mean age, and baseline LVEF.

### Quality assessment and certainty of evidence

The risk of bias within individual studies was assessed using the Newcastle-Ottawa Quality Assessment Scale for Cohort Studies. Initial evaluations were conducted by J.S.V. and subsequently verified by A.D.M.

The certainty of evidence for each outcome was assessed using the GRADE (Grading of Recommendations Assessment, Development and Evaluation) framework,^13^ according to risk of bias, inconsistency, indirectness, imprecision, and publication bias.

### Statistical analysis

Studies were grouped according to the reported outcome (CRT response or arrhythmic events) for each synthesis. Binary outcomes from individual studies were pooled using a random-effects model with restricted maximum likelihood to estimate ORs and 95% CIs. A continuity correction was applied for studies with zero events in any group. Statistical significance was defined as a 2-sided *P* value <0.05. Forest plots were used to visually display individual study estimates and pooled effects.

Heterogeneity between studies was assessed using Cochran Q test (*P* < 0.10 considered significant) and quantified with the I^2^ statistic, which describes the percentage of variability in effect estimates due to heterogeneity rather than chance. I^2^ values of 25%, 50%, and 75% were considered to represent low, moderate, and high heterogeneity, respectively. Publication bias was evaluated using the Egger and Peters tests (both significant at *P* < 0.10). Subgroup and sensitivity analyses were performed according to study design, LGE assessment method, endpoint definition, restriction to studies reporting both endpoints, and exclusion of the smallest studies. All analyses were conducted using Stata (version 17, StataCorp).

## Results

### Search results and study selection

The search identified 1793 references. After duplicate removal, 1,046 records were screened by title and abstract, of which 996 were excluded, leaving 50 for full-text review. Of these, 34 did not meet the inclusion criteria (see Supplemental Refs. 1-34). The main reasons for the exclusion of the manuscripts were the inclusion of both ischemic and nonischemic patients (without providing specific data on NICM) and the analysis limited to LGE quantification without providing data about the prognostic role of the simple dichotomous variable LGE present/absent (Supplemental Table 3). In addition, 6 studies were excluded^6^^,^^14^, ^15^, ^16^, ^17^, ^18^ because their data were included in more recent publications.^19^, ^20^, ^21^

Ultimately, 10 studies were included in the meta-analysis. Of these, 6 contributed to the analysis of the arrhythmic endpoint,^10^^,^^11^^,^^19^^,^^22^, ^23^, ^24^ 9 assessed the response to CRT^9^, ^10^, ^11^^,^^20^, ^21^, ^22^, ^23^, ^24^, ^25^ (Figure 1).

**Figure 1:**
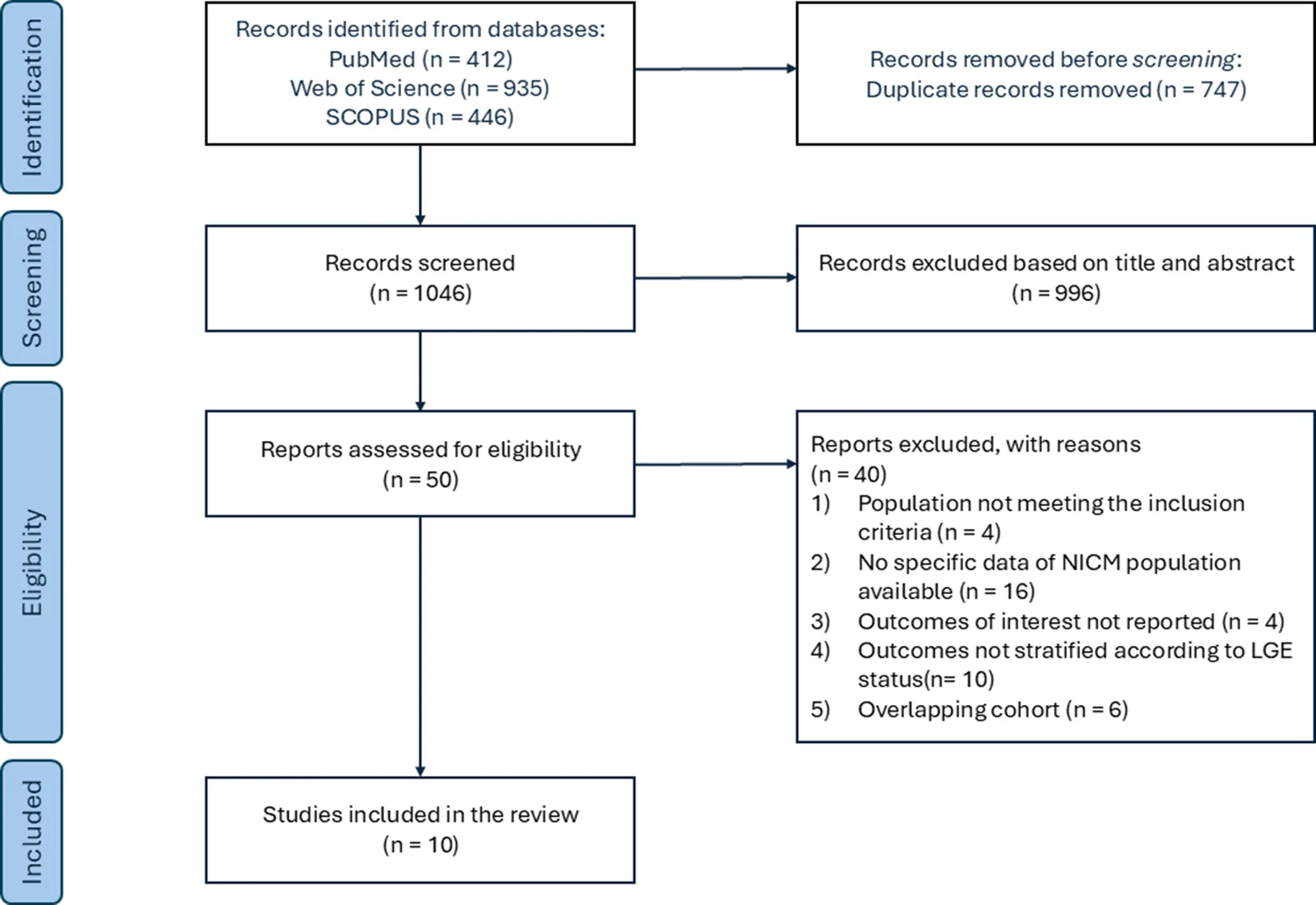
PRISMA Diagram PRISMA flow diagram showing the identification, screening, eligibility assessment, and inclusion of studies included in the meta-analysis. LGE = late gadolinium enhancement; NICM = nonischemic cardiomyopathy.

### Study quality and certainty of evidence

The Newcastle-Ottawa Quality Assessment Scale revealed that, in many cases, the comparability of study groups was not fully ensured due to the observational nature of all studies and the lack of multivariate analysis in many of them. Despite this limitation, most reports fulfilled all other criteria (Supplemental Tables 4 and 5).

According to the GRADE framework, the certainty of evidence was initially rated as low for both outcomes because all included studies were observational. The certainty of evidence for VAs was subsequently upgraded to moderate owing to the large magnitude of the observed association.

### Study characteristics

A total of 1,085 patients from 10 studies were included in the meta-analysis: 831 patients from 9 studies for the echocardiographic response endpoint and 745 patients from 6 studies for the arrhythmic endpoint. All studies included patients with class I or II indication for CRT according to the corresponding guidelines.^2^^,^^26^ Three studies included only patients with left bundle branch block,^9^^,^^11^^,^^22^ and 2 included only those in sinus rhythm.^22^^,^^25^ CRT was obtained by left bundle branch area pacing in 1 study^11^ and by traditional biventricular pacing in the rest. The mean baseline LVEF ranged from 20% to 31% across studies, and follow-up duration ranged from 21 to 83 months. Study characteristics and endpoint definitions are summarized in Tables 1 and 2, and additional methodological details (NICM definition and LGE assessment) are provided in Supplemental Tables 6 and 7.

**Table 1:** Baseline Characteristics of Included Studies

First Author, Year	Type of Study	n	Population Considered for Meta-Analysis	Mean Age (y)	Mean LVEF (%)	LGE+	Median Follow-Up
Fernández-Armenta, 2012^23^	Prospective cohort, consecutive patients	36^a^	NICM, primary prevention CRT-D, QRSd ≥120 ms and/or LV dyssynchrony	64^b^	22^b^	15 (42%)^a^	25 months^b^
Andre, 2018^22^	Prospective cohort, consecutive patients	36^a^	NICM, primary prevention CRT-D, LBBB, QRSd ≥120 ms, SR, NYHA functional class II-III	61^b^	27^b^	11 (31%)^a^	37 months^b^ (mean)
Linhart, 2020^24^	Prospective cohort, consecutive patients	138^a^	NICM, CRT-P or primary prevention CRT-D, QRSd ≥120 ms, NYHA class II-III, ambulatory IV	64^b^	22^b^	62 (45%)^a^	45 months^b^
Leyva, 2022^19^	Prospective observational study	255^a^	NICM, CRT-P or primary prevention CRT-D according to NICE guidelines	68^b^	28^b^	64 (25%)^a^	6.93 years^b^
Duchenne, 2023^21^	Prospective observational study	91^a^	NICM, CRT	67^b^	31^b^	30 (33%)^a^	1,543 days^b^
Maffessanti, 2023^25^	Prospective observational study	12^a^	NICM, CRT, QRSd >120 ms, LBBB and/or intraventricular conduction delay, SR	NI	25^b^	7 (58%)^a^	63 months^b^
Ponnusamy, 2023^11^	Prospective cohort, consecutive patients	116	NICM, LBBB, LBBP in CRT-eligible patients, QRSd >140 ms (male)/>130 ms (female)	58	30	22 (19%)	21 months
Song, 2023^9^	Retrospective cohort study, consecutive patients	98	NICM, LBBB, CRT	59	20	33 (34%)	2.5 years (mean)
Ma, 2024^20^	Retrospective observational study	141^a^	NICM, CRT	57^b^	31^b^	90 (64%)^a^	1,543 days^b^
Anguera, 2025^10^	Retrospective cohort study, consecutive patients	167	NICM, CRT-P or primary prevention CRT-D, LBBB with QRSd >130 ms or QRSd >150 ms without LBBB	64	25	77 (46%)	63 months

CRT = cardiac resynchronization therapy; CRT-D = CRT with defibrillator; CRT-P = CRT with pacemaker; LBBB = left bundle branch block; LBBP = left bundle branch pacing; LGE = late gadolinium enhancement; LV = left ventricular; LVEF = left ventricular ejection fraction; NI = not informed; NICM = nonischemic cardiomyopathy; QRSd = QRS duration; SR = sinus rhythm.

a The population included in this meta-analysis corresponds to a subset of the total study population.

b Data obtained from the overall study population.

**Table 2:** Definitions of Study Endpoints

First Author, Year	Response to CRT	Arrhythmic Endpoints Evaluated
Fernández-Armenta, 2012^23^	Composite criterion: ≥15% reduction in LVESV at 12 months and survival free of heart transplant	ICD therapies
Andre, 2018^22^	Composite criterion: ≥15% reduction in LVESV at 6 months and no death or hospitalization for major adverse cardiac events (MACE: HF hospitalization, cardiogenic shock, sustained VT)	Hospitalization for sustained VT
Linhart, 2020^24^	≥15% reduction in LVESV at 12 months	SCD, sustained VT, ICD therapies
Leyva, 2022^19^	NA	SCD, resuscitated cardiac arrest, sustained VT, VF, ICD therapies
Duchenne, 2023^21^	≥15% reduction in LVESV at 12 months	NA
Maffessanti, 2023^25^	≥15% reduction in LVESV at 6 months	NA
Ponnusamy, 2023^11^	Absolute increase in LVEF ≥15% at 6 months	SCD, sustained VT, ICD therapies
Song, 2023^9^	Relative increase in LVEF ≥15% at 6 months	NA
Ma, 2024^20^	Composite criterion: Absolute increase in LVEF ≥5% and improvement of ≥1 NYHA functional class at 6 months	NA
Anguera, 2025^10^	Improvement in LVEF ≥10% at 6–12 months	SCD, resuscitated cardiac arrest, sustained VT, ICD therapies

HF = heart failure; ICD = implantable cardioverter-defibrillator; LVESV = left ventricular end-systolic volume; MACE = major adverse cardiac events; NA = not assessed in the study; SCD = sudden cardiac death; VF = ventricular fibrillation; VT = ventricular tachycardia; other abbreviations as in Table 1.

### LGE and CRT response

In the 9 studies evaluated, the prevalence of LGE among patients with NICM ranged from 20% to 64%, with an overall pooled prevalence of 41.8% (347 patients). CRT response occurred in 557 patients (67.0%), including 176 of 347 (50.7%) patients with LGE (LGE+) and 381 of 484 (78.7%) cases without LGE (LGE−), as summarized in Table 3. The weighted risk difference between LGE- and LGE+ patients was 24.8% (95% CI: 13.8% to 35.7%; *P* < 0.001). The absence of LGE was associated with a significantly higher likelihood of CRT response, with a pooled OR of 3.52 (95% CI: 2.54-4.88; *P* < 0.001) (Figure 2).

**Table 3:** Echocardiographic Response and Ventricular Arrhythmias According to the Presence of Late Gadolinium Enhancement

First Author, Year	% of Patients With Echocardiographic Response			% of Patients With Ventricular Arrhythmias		
	LGE−	LGE+	*P* Value	LGE−	LGE+	*P* Value
Fernández-Armenta, 2012^23^	71.4%	73.3%	1.000	0.0%	46.7%	0.002
Andre, 2018^22^	84.0%	90.9%	0.977	0.0%	0.0%	1.000
Linhart, 2020^24^	72.4%	50.0%	0.012	1.3%	25.8%	<0.001
Leyva, 2022^19^	-	-	-	1.6%	12.5%	<0.001
Duchenne, 2023^21^	91.8%	73.3%	0.041	-	-	-
Maffessanti, 2023^25^	100%	42.9%	0.147	-	-	
Ponnusamy, 2023^11^	70.0%	36.4%	0.007	0.0%	13.6%	0.007
Song, 2023^9^	87.7%	42.4%	<0.001	-	-	-
Ma, 2024^20^	70.6%	40.0%	<0.001	-	-	-
Anguera, 2025^10^	81.1%	53.2%	<0.001	0.0%	24.7%	<0.001
Overall	78.7%	50.7%	<0.001	0.8%	21.1%	<0.001

Values represent proportions of patients with echocardiographic response or ventricular arrhythmias according to the presence or absence of LGE. *P* values were calculated using the chi-square or Fisher exact test (when cell counts <5).

Abbreviation as in Table 1.

**Figure 2:**
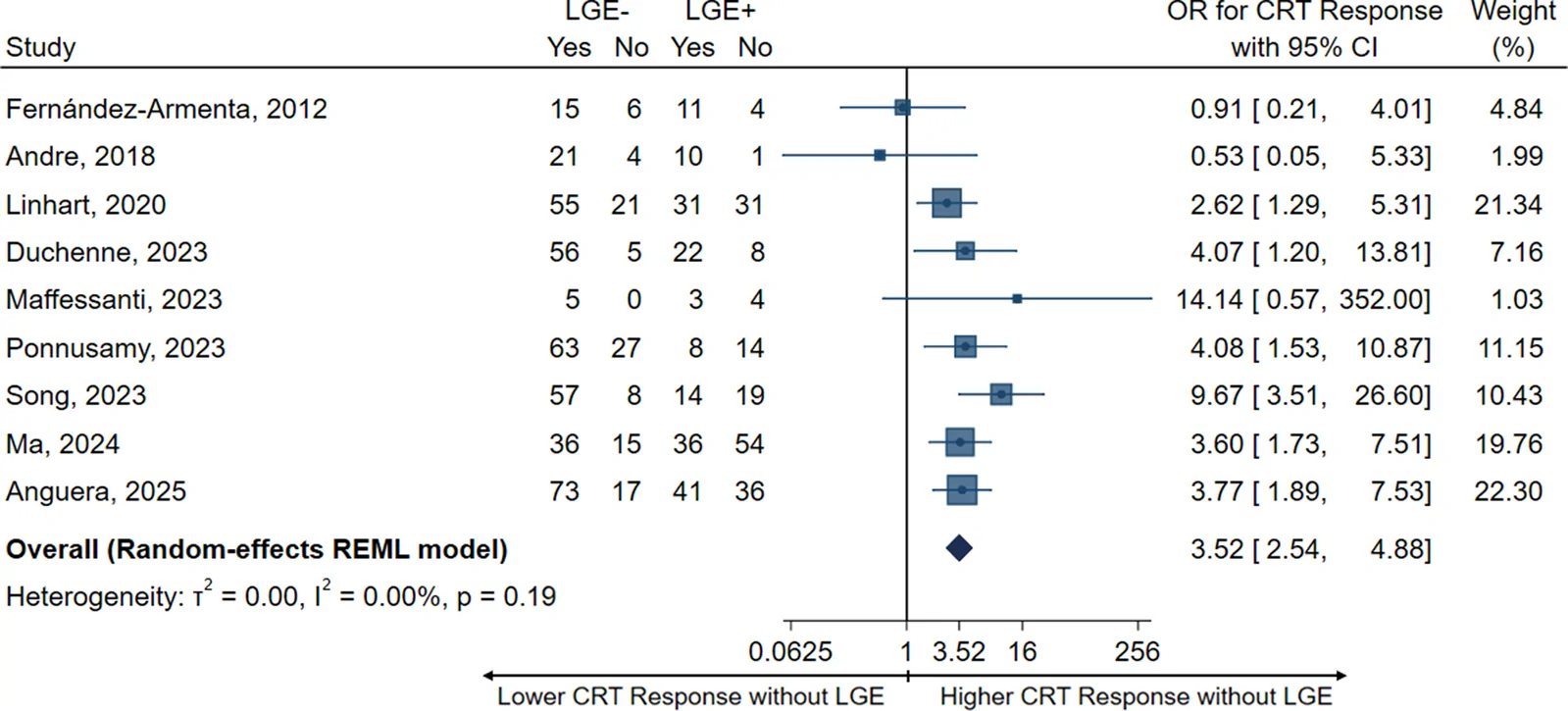
Forest Plot of Cardiac Resynchronization Therapy Response ORs for echocardiographic response to CRT according to the presence or absence of LGE. Squares represent individual study estimates (size proportional to weight), horizontal lines the 95% CI and the diamond the pooled estimate from a random-effects model. CRT = cardiac resynchronization therapy; REML = restricted maximum likelihood; other abbreviation as in Figure 1.

Heterogeneity was low (I^2^ = 0%; *P* = 0.191), and results were consistent across fixed- and random-effects models (Supplemental Table 8). Egger and Peters tests suggested the absence of publication bias (Supplemental Table 9), consistent with the funnel plot analysis (Supplemental Figure 1).

### LGE and arrhythmias

In the 6 works evaluated, LGE was present in 251 patients (34%), with proportion across studies ranging from 20% to 45%. The VA endpoint occurred in 57 patients (7.7%), including 53 of 251 (21.1%) LGE+ patients and only 4 of 494 (0.8%) LGE− ones, as summarized in Table 3. Notably, among the 494 LGE− patients not a single sudden death occurred, the 4 VA events being all nonfatal.^19^^,^^24^ In addition, in most studies (4 of 6), zero arrhythmic events were observed in LGE− patients.^10^^,^^11^^,^^22^^,^^23^

The mean follow-up in the 6 studies included in this analysis was 57 months. The average annual incidence of VA was 0.17 every 100 patients/year (95% CI: 0.05-0.43) in LGE− patients and 4.48 every 100 patients/year (95% CI: 3.36-5.86) in LGE+ ones. Therefore, the estimated 5-year incidence of VA and sudden cardiac death was 0.84% in LGE− cases and 20.1% in LGE+ ones.

The weighted risk difference between LGE+ and LGE− patients was 17.5% (95% CI: 6.3%-28.6%; *P* = 0.002). The absence of LGE was associated with a significantly lower risk of arrhythmic events, with a strikingly low pooled OR of 0.06 (95% CI: 0.02-0.15; *P* < 0.001) (Figure 3).

**Figure 3:**
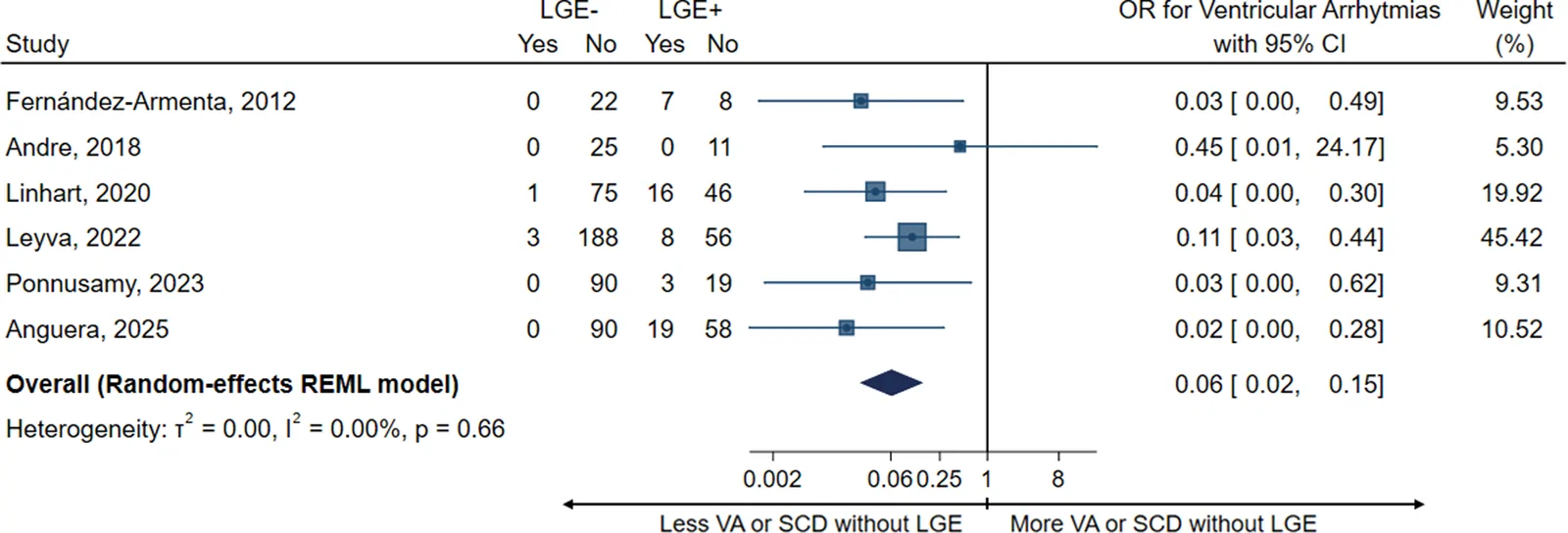
Forest Plot of Ventricular Arrhythmic Events ORs for ventricular arrhythmic events or sudden cardiac death according to the presence or absence of LGE. Squares indicate individual study estimates (size proportional to weight), horizontal lines the 95% CI, and the diamond the pooled estimate from a random-effects model. SCD = sudden cardiac death; VA = ventricular arrhythmia; other abbreviations as in Figures 1 and 2.

Heterogeneity was negligible (I^2^ = 0%; *P* = 0.656), and results were consistent across fixed- and random-effects models (Supplemental Table 10). Egger and Peters tests suggested the absence of publication bias (Supplemental Table 9), consistent with the funnel plot analysis (Supplemental Figure 2).

### Subgroup analysis and sensitive analysis

We performed subgroup analyses to evaluate the consistency of the association between absence of LGE and the 2 clinical endpoints (Supplemental Tables 11 and 12).

With respect to echocardiographic response to CRT, the absence of LGE was consistently associated with a higher likelihood of response in both prospective^11^^,^^21^, ^22^, ^23^, ^24^, ^25^ (OR: 2.71; 95% CI: 1.68-4.35) and retrospective studies^9^^,^^10^^,^^20^ (OR: 4.51; 95% CI: 2.80-7.26). The association was also maintained both in studies defining response as LVEF improvement^9^, ^10^, ^11^^,^^20^ (OR: 4.40; 95% CI: 2.92-6.63) and in those which employed left ventricular end-systolic volume reduction^21^, ^22^, ^23^, ^24^, ^25^ (OR: 2.38; 95% CI: 1.39-4.10). Similar results were observed in studies using visual assessment of LGE^9^, ^10^, ^11^^,^^20^^,^^22^^,^^24^ (OR: 3.69; 95% CI: 2.60-5.24). No statistically significant differences were observed between subgroups according to study design, LGE assessment method, or response definition (*P* for subgroup differences = 0.136, 0.645, and 0.077, respectively).

For the arrhythmic endpoint, the absence of LGE was significantly associated with a lower risk of events in prospective^11^^,^^19^^,^^22^, ^23^, ^24^ (OR: 0.07; 95% CI: 0.03-0.19) and retrospective studies^10^ (OR: 0.02; 95% CI: 0.00-0.28) and in those using visual^10^^,^^11^^,^^19^^,^^22^^,^^24^ (OR: 0.07; 95% CI: 0.03-0.18) or automated assessment of LGE^23^ (OR: 0.03; 95% CI: 0.00-0.49). No statistically significant differences were observed according to study design or LGE assessment method (*P* for subgroup differences = 0.338 and 0.538, respectively).

In the study by Ponnusamy et al,^11^ patients with ≥10% fibrosis were systematically implanted with CRT-D, whereas most LGE- patients received CRT-P. Because arrhythmic events were defined as ICD therapies, this design may have introduced ascertainment bias. A sensitivity analysis excluding this study confirmed that the absence of LGE remained significantly associated with a lower risk of VA or sudden death (OR: 0.07; 95% CI: 0.03-0.17).

After excluding the smallest studies (total sample size ≤36 patients),^22^^,^^23^^,^^25^ the absence of LGE remained significantly associated with a higher likelihood of echocardiographic response to CRT (OR: 3.88; 95% CI: 2.76-5.45) and a lower risk of VAs (OR: 0.06; 95% CI: 0.02-0.16).

Finally, when restricting the analysis to the 5 studies that assessed both endpoints,^10^^,^^11^^,^^22^, ^23^, ^24^ the absence of LGE remained significantly associated with higher CRT response (OR: 2.89; 95% CI: 1.87-4.29) and lower risk of VA (OR: 0.04; 95% CI: 0.01-0.13).

### Analysis adjusting for confounding covariates

Two studies including a total of 266 patients reported adjusted estimates for echocardiographic response to CRT.^9^^,^^10^ After controlling for potential confounders, the pooled adjusted OR was 4.19 (95% CI: 2.18-8.04), confirming that the absence of LGE is an independent predictor of CRT response (Table 4).

**Table 4:** Multivariable-Adjusted ORs for Echocardiographic Response to Cardiac Resynchronization Therapy According to the Absence of Late Gadolinium Enhancement

First Author, Year	Adjusted Covariates	Adjusted Response to CRT OR (95% CI)	Pooled OR (95% CI), *P* Value
Song, 2023^9^	Absence of LGE, QRS duration, LVEF, circumferential time to peak SD	7.14 (2.13-25.00)	4.19 (2.18-8.04), *P* < 0.001
Anguera, 2025^10^	Absence of LGE, no history of AF, absence of first-degree AVB, Ventricular pacing (%)	3.4 (1.6-7.4)	

Pooled estimate calculated using a random-effects model with restricted maximum likelihood (REML).

AF = atrial fibrillation; AVB = atrioventricular block; other abbreviations as in Table 1.

In addition, the study that performed a multivariable analysis for the arrhythmic endpoint identified the absence of LGE as a strong and independent predictor of lower arrhythmic risk (HR: 0.04; 95% CI: 0.00-0.33), after adjustment for QRS duration and CRT response.^10^

## Discussion

This meta-analysis, including 1,085 patients from 10 studies, is the first systematic review to specifically include only NICM patients implanted with a CRT and to assess the association between LGE absence and both echocardiographic response and the risk of VAs. The results demonstrate that the absence of LGE is associated with a significantly higher likelihood of echocardiographic response to CRT and a very low risk of VAs (Central Illustration).

**Central Illustration:**
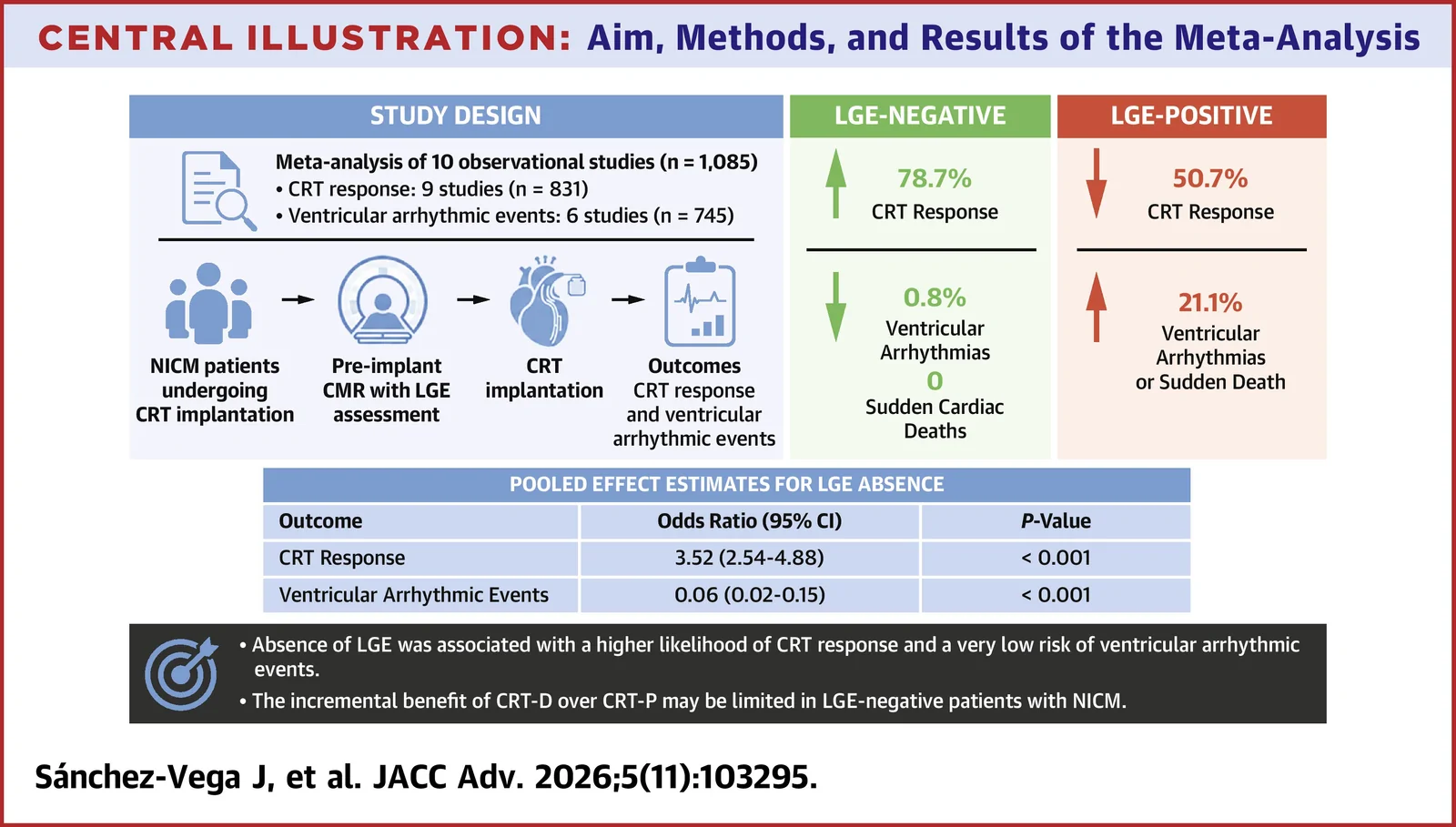
Aim, Methods, and Results of the Meta-Analysis A meta-analysis of observational studies evaluating cardiac resynchronization therapy (CRT) in patients with nonischemic cardiomyopathy (NICM), stratified by the presence of late gadolinium enhancement (LGE). The absence of LGE was associated with a higher likelihood of echocardiographic response and markedly lower incidence of ventricular arrhythmias and sudden death, supporting CRT-P indication in LGE-cases. CMR = cardiac magnetic resonance; CRT = cardiac resynchronization therapy; CRT-D = cardiac resynchronization therapy with defibrillator; CRT-P = cardiac resynchronization therapy pacemaker; LGE = late gadolinium enhancement; NICM = nonischemic cardiomyopathy.

### Prior studies

No meta-analysis has previously focused on the prognostic role of LGE in NICM patients with CRT.

One meta-analysis^27^ evaluated the association between LGE quantification and response to CRT, including studies with mixed populations of ischemic and NICM patients, but had overtly no statistical power to evaluate the subgroup of NICM patients.

Another meta-analysis^28^ evaluated the association between LGE presence and sudden cardiac death only (not including VA) in CRT recipients with ischemic heart disease or NICM. Again, no specific data about NICM patients were provided.

We strongly believe that both the risk prediction for VA/sudden death and the prediction of response to CRT should be evaluated separately in ischemic and NICM patients, as they are different entities, with different risk profile and potentially different risk factors. The relevance of analyzing separately ischemic and nonischemic patients is even greater when assessing the prognostic role of LGE, as LGE is present in virtually all patients with ischemic heart disease and severe left ventricular dysfunction, although it is observed in just about 40% of NICM patients.

Notably, one of the main reasons for exclusion of studies in this meta-analysis was actually that they reported mixed data on ischemic and nonischemic patients, without providing specific results for the 2 groups of patients. These considerations have important implications for future studies evaluating outcome in CRT: such works should definitely analyze separately ischemic and nonischemic patients.

### Clinical implications

No clinical trial has demonstrated a survival benefit from primary prevention ICD in NICM with LVEF ≤35%. Theoretically, the need for an ICD might be even lower in patients implanted with a CRT, as CRT per se has a significant impact on prognosis.^29^ A subanalysis of 555 NICM patients included in the COMPANION trial showed significantly reduced mortality with CRT-D as compared to CRT-P.^30^ However, this trial included patients between 2000 and 2002, when the medical treatment for heart failure was not comparable to current one and the incidence of sudden death in heart failure was considerably higher than nowadays.^31^ In fact, the more recent DANISH trial found no significant survival differences between CRT-D and CRT-P in 646 patients with NICM.^32^ Actually, in the long-term follow-up of the DANISH trial, a significant reduction in sudden death with ICD was observed in patients without CRT but not in those with CRT.^33^ One meta-analysis, including these 2 randomized trials, did not observe a survival benefit of CRT-D vs CRT-P in NICM.^1^

In summary, prior evidence suggests that CRT-P may be actually enough for NICM as a whole; however, this observation comes into contradiction with the fact that most of patients with class I indication for CRT also have a class IIa indication for primary prevention ICD.^2^^,^^3^ Therefore, it is urgent to identify which subgroups of patients can be safely implanted with a CRT-P and which truly need a CRT-D.

Several studies, also summarized in various meta-analysis, have found that LGE is a strong and independent predictor of VA and sudden death in NICM.^4^^,^^5^^,^^34^ This is consistent with the role of myocardial fibrosis as a key arrhythmogenic substrate facilitating reentry.^35^ Two relevant aspects of these works were that: 1) the simple dichotomous discrimination between LGE+ and LGE- cases had great prognostic relevance; and 2) the absence of LGE was associated with a high negative predictive value for VA and sudden death.

These results are confirmed and even amplified in CRT recipients, according to our meta-analysis. The combination of baseline low arrhythmic risk and high probability of a significant improvement in LVEF likely contributes to the extremely low arrhythmic risk in LGE- NICM recipients of CRT. In fact, one should remember that CRT response per se is also associated with a diminished risk of VA.^36^

In a total of 494 LGE- patients with CRT, the average annual incidence of VA or sudden death was 0.17%, implying that the estimated 5-year incidence was lower than 1% (0.84%). Notably, these numbers overestimate the true incidence of sudden death, as not all VA are lethal: notably, no sudden death at all was observed in any of the included study in LGE- patients. In the DANISH trial appropriate ICD therapies occurred at least 4 times more frequently than sudden death preventable by ICD.^32^ Therefore, the 5-year risk of sudden death preventable with an ICD in LGE- patients with CRT could be as low as 0.2%: we should implant 500 CRT-D in LGE- patients to save one life in a 5-year period. With such a low arrhythmic risk, it is unlikely that primary prevention ICD will have a significant impact on overall mortality in LGE- CRT candidates with NICM. These data can also explain why an observational study found that, in NICM, CRT-D was superior to CRT-P in LGE+ cases but not in LGE- ones.^16^

In the absence of specifically designed randomized trials, clinical guidelines may be influenced by the results of observational studies, especially if they show strong and consistent associations.

For example, the last guidelines of the European Society of Cardiology for the management of VA recommended primary prevention ICD in NICM patients with LVEF>35% and at least 2 risk factors for sudden death (LGE, syncope, high risk genetics, or inducible ventricular tachycardia).^37^ This was a pragmatical approach based only on the evidence from observational studies.

In a context where subgroup analysis of randomized trials already suggest no mortality benefit of CRT-D over CRT-P in NICM,^1^ the results of this meta-analysis, despite originating from observational studies, may contribute to refine guidelines recommendations underscoring that the incremental benefit of CRT-D may be limited especially in NICM patients without LGE, at least while we await the results of clinical trials such as the SPANISH trial (NCT06055504), which will help clarify the role of ICD therapy in patients with NICM without LGE, including those who are candidates for CRT.

Notably, among NICM patients with primary prevention indication to ICD due LVEF ≤35%, those who also have an indication to CRT are the majority (for example, 58% of patients included in the DANISH trial had CRT). In addition, these patients, as compared to those candidates to single or dual chamber ICD, receive an additional treatment (CRT) which can substantially improve LVEF and has a strong prognostic impact. Therefore, future guidelines dealing with primary prevention ICD indications should provide separate recommendations in NICM patients depending on whether they will receive CRT or not.

One should keep in mind that adding a defibrillator to CRT implies relevant costs, both economical and in terms of potential complications for patients. The cost of CRT-D devices is at least the double of CRT-P ones; this economical gap is even wider if we consider that left bundle branch area pacing allows cardiac resynchronization with a standard dual-chamber pacemaker. In addition, the longevity of CRT-D is shorter than CRT-P and much shorter than dual-chamber pacemakers,^38^ further increasing the costs of adding a defibrillator and implying greater risks for patients due to higher need of battery replacements. Finally, CRT-D is associated with a risk of inappropriate shocks, which can produce significant stress to patients. Therefore, a more appropriate selection of candidates to CRT-D may have important clinical and economical implications.

The pooled analysis showed that LGE+ patients had a significantly higher risk of VA and sudden death and may therefore benefit from primary prevention CRT-D. However, further studies are needed to refine the arrhythmic risk stratification even in LGE+ cases.

### Implications for future studies

In the present meta-analysis we had to exclude several studies because, despite evaluating the predictive role of CMR in CRT, they only analyzed LGE quantification and not the simple dichotomous variable LGE presence/absence (Supplemental Refs. 25-33). LGE quantification might be important to refine the risk stratification among LGE + patients. However, the results of this meta-analysis show that the simple distinction between LGE presence and absence has, per se, strong prognostic implications in CRT recipients with NICM. Thus, it is highly advisable that future studies evaluating outcomes in patients with CRT and NICM report also on the impact of LGE presence/absence.

Traditionally the literature has paid more attention to the increased arrhythmic risk associated with LGE presence than the low arrhythmic risk associated with LGE absence. With this manuscript we suggest that shifting the focus toward LGE absence may provide clinically relevant data: in this case, it allowed the identification of a subgroup of NICM patients who may not deserve a primary prevention CRT-D.

### Study limitations

The main limitation of this meta-analysis is the inclusion of observational studies, which share an inherent risk of selection bias and preclude causal inference. Although the main results are based on pooled unadjusted ORs, multivariable analysis, when performed, consistently demonstrated that the absence of LGE is an independent predictor of CRT response and of low arrhythmic risk, and subgroup analyses yielded consistent results. Definitions of both echocardiographic response and arrhythmic outcomes varied across studies. The lack of standardized criteria for CRT response^39^ may have introduced outcome misclassification and clinical heterogeneity across studies, potentially influencing the effect estimates; however, subgroup analyses according to response definition yielded consistent results. Similarly, the arrhythmic endpoint introduced clinical heterogeneity by combining distinct events, including sustained ventricular tachycardia, appropriate ICD therapies, resuscitated cardiac arrest, and sudden cardiac death. Because sustained VAs and ICD therapies do not necessarily represent life-threatening events, their inclusion may overestimate the true risk of sudden cardiac death. Nevertheless, the very low incidence of arrhythmic events and the absence of sudden death among LGE- patients support the overall findings. Some studies also had small sample sizes, potentially reducing the precision of individual effect estimates; however, sensitivity analyses excluding the smallest studies yielded results consistent with the primary analysis, suggesting that the pooled findings were not driven by the smallest cohorts. Genetic analysis was not detailed in the included studies. Although genetic is an important predictor of outcome, the meta-analysis provides strong observational evidence about the extremely low arrhythmic risk in LGE- patients treated with CRT. With such a low risk, close to zero, it is unlikely that any other confounder can significantly modify this scenario.

In addition, variability in LGE analysis and reporting limited our ability to examine how different patterns or the extent of fibrosis modulate arrhythmic risk or CRT response in LGE+ patients.

Finally, the review protocol was not prospectively registered in PROSPERO, although the review process and reporting were aligned with the PRISMA 2020 recommendations.

## Conclusions

In patients with NICM undergoing CRT implant, the absence of LGE is strongly associated with greater echocardiographic response and a very low risk of VAs. These findings may have important implications in clinical practice, suggesting that the incremental benefit of CRT-D over CRT-P may be limited in NICM patients without LGE.

Perspectives **COMPETENCY IN MEDICAL KNOWLEDGE:** The absence of LGE on CMR is associated with significantly greater CRT response and a very low risk of VAs in NICM patients.

**TRANSLATIONAL OUTLOOK:** NICM patients without LGE and without a history of sustained VAs, who are candidates to CRT, may represent a subgroup in whom the incremental benefit of adding a defibrillator to CRT is limited.

## Funding support and author disclosures

The authors have reported that they have no relationships relevant to the contents of this paper to disclose.
