# Supplementary material for: Absence of Fibrosis Influences Prognosis of Cardiac Resynchronization in Nonischemic Cardiomyopathy: Systematic Review and Meta-Analysis
Source: JACC Adv. 2026 Oct 1;5(11):103295. doi: 10.1016/j.jacadv.2026.103295 (PMC13653926; doi:10.1016/j.jacadv.2026.103295)
Supplement: Supplementary file 1 [file mmc1.pdf]

SUPPLEMENTAL APPENDIX

Supplemental Tables **Pages**

Supplemental Table1. PRISMA 2020 Checklist……………………………………………….3

Supplemental Table 2. Detailed search strategy………………………………………………..5

Supplemental Table 3. List of manuscripts excluded after full text revision and reasons for exclusion……………………………………………………………………………………..6

Supplemental Table 4. Newcastle–Ottawa Scale (NOS) Quality Assessment of Included Studies Evaluating Echocardiographic Response to CRT……………………………………………7

Supplemental Table 5. Newcastle–Ottawa Scale (NOS) Quality Assessment of Included Studies Evaluating Ventricular Arrhythmic Events……………………………………………………8

Supplemental Table 6. Criteria to define nonischemic cardiomyopathy in each study ……..…..9

Supplemental Table 7. Methods for evaluation of late gadolinium enhancement in included studies ……………………………………………………………………………………..….10

Supplemental Table 8. Odds ratio for CRT response using fixed- and random-effects models...11

Supplemental Table 9. Analysis of publication bias…………………………………..……12

Supplemental Table 10. Odds ratio for ventricular arrhythmias using fixed- and random-effects models ………………………………………………………………………………………...13

Supplemental Table 11. Subgroup analyses of the association between LGE and echocardiographic response to CRT………………………………………………………..14

Supplemental Table 12. Subgroup analyses of the association between LGE and ventricular arrhythmic events……………………………………………………………………………...15

Supplemental Figures

Supplemental Figure 1. Funnel plot of CRT response………………………………………. 16

Supplemental Figure 2. Funnel plot of ventricular arrhythmic events ...……………………….17

References

Supplemental References………………………………………………………………...18

SUPPLEMENTAL TABLES

Supplemental Table 1. PRISMA 2020 Checklist

Section and Topic	Item #	Checklist item	Location where item is reported
**TITLE**			
Title	1	Identify the report as a systematic review.	Title
**ABSTRACT**			
Abstract	2	See the PRISMA 2020 for Abstracts checklist.	Abstract
**INTRODUCTION**			
Rationale	3	Describe the rationale for the review in the context of existing knowledge.	Page 5, Line 2-9
Objectives	4	Provide an explicit statement of the objective(s) or question(s) the review addresses.	Page 5, Line 21-23
**METHODS**			
Eligibility criteria	5	Specify the inclusion and exclusion criteria for the review and how studies were grouped for the syntheses.	Line 42-51
Information sources	6	Specify all databases, registers, websites, organisations, reference lists and other sources searched or consulted to identify studies. Specify the date when each source was last searched or consulted.	Line 32-34
Search strategy	7	Present the full search strategies for all databases, registers and websites, including any filters and limits used.	Supplemental Table 2<br>Line 35-39
Selection process	8	Specify the methods used to decide whether a study met the inclusion criteria of the review, including how many reviewers screened each record and each report retrieved, whether they worked independently, and if applicable, details of automation tools used in the process.	Line 64-68
Data collection process	9	Specify the methods used to collect data from reports, including how many reviewers collected data from each report, whether they worked independently, any processes for obtaining or confirming data from study investigators, and if applicable, details of automation tools used in the process.	Line 69-71
Data items	10a	List and define all outcomes for which data were sought. Specify whether all results that were compatible with each outcome domain in each study were sought (e.g. for all measures, time points, analyses), and if not, the methods used to decide which results to collect.	Line 54-57
	10b	List and define all other variables for which data were sought (e.g. participant and intervention characteristics, funding sources). Describe any assumptions made about any missing or unclear information.	Line 72-74
Study risk of bias assessment	11	Specify the methods used to assess risk of bias in the included studies, including details of the tool(s) used, how many reviewers assessed each study and whether they worked independently, and if applicable, details of automation tools used in the process.	Line 77-79
Effect measures	12	Specify for each outcome the effect measure(s) (e.g. risk ratio, mean difference) used in the synthesis or presentation of results.	Line 86-66
Synthesis methods	13a	Describe the processes used to decide which studies were eligible for each synthesis (e.g. tabulating the study intervention characteristics and comparing against the planned groups for each synthesis (item #5)).	Line 85-86
	13b	Describe any methods required to prepare the data for presentation or synthesis, such as handling of missing summary statistics, or data conversions.	Line 88-89
	13c	Describe any methods used to tabulate or visually display results of individual studies and syntheses.	Line 89-90
	13d	Describe any methods used to synthesize results and provide a rationale for the choice(s). If meta-analysis was performed, describe the model(s), method(s) to identify the presence and extent of statistical heterogeneity, and software package(s) used.	Line 86-99
	13e	Describe any methods used to explore possible causes of heterogeneity among study results (e.g. subgroup analysis, meta-regression).	Line 91-95
	13f	Describe any sensitivity analyses conducted to assess robustness of the synthesized results.	Line 95-98
Reporting bias assessment	14	Describe any methods used to assess risk of bias due to missing results in a synthesis (arising from reporting biases).	Line 94-95
Certainty assessment	15	Describe any methods used to assess certainty (or confidence) in the body of evidence for an outcome.	Line 80-82

Section and Topic	Item #	Checklist item	Location where item is reported
**RESULTS**			
Study selection	16a	Describe the results of the search and selection process, from the number of records identified in the search to the number of studies included in the review, ideally using a flow diagram.	Figure 1<br>Line 103-110
	16b	Cite studies that might appear to meet the inclusion criteria, but which were excluded, and explain why they were excluded.	Supplemental Table 3
Study characteristics	17	Cite each included study and present its characteristics.	Table 1<br>Line 111-113
Risk of bias in studies	18	Present assessments of risk of bias for each included study.	Supplemental Tables 4 and 5<br>Line 116-119
Results of individual studies	19	For all outcomes, present, for each study: (a) summary statistics for each group (where appropriate) and (b) an effect estimate and its precision (e.g. confidence/credible interval), ideally using structured tables or plots.	Table 3
Results of syntheses	20a	For each synthesis, briefly summarise the characteristics and risk of bias among contributing studies.	Table 2<br>Supplemental Tables 6 and 7<br>Line 126-135
	20b	Present results of all statistical syntheses conducted. If meta-analysis was done, present for each the summary estimate and its precision (e.g. confidence/credible interval) and measures of statistical heterogeneity. If comparing groups, describe the direction of the effect.	Figures 2–3<br>Line 138-169
	20c	Present results of all investigations of possible causes of heterogeneity among study results.	Supplemental Tables 11 and 12<br>Line 145-148, 166-200
	20d	Present results of all sensitivity analyses conducted to assess the robustness of the synthesized results.	Supplemental Tables 11 and 12<br>172-200
Reporting biases	21	Present assessments of risk of bias due to missing results (arising from reporting biases) for each synthesis assessed.	Supplemental Figures 1 and 2<br>Supplemental Table 9
Certainty of evidence	22	Present assessments of certainty (or confidence) in the body of evidence for each outcome assessed.	Line 120-123
**DISCUSSION**			
Discussion	23a	Provide a general interpretation of the results in the context of other evidence.	Line 214-219
	23b	Discuss any limitations of the evidence included in the review.	Line 336-363
	23c	Discuss any limitations of the review processes used.	Line 336-363
	23d	Discuss implications of the results for practice, policy, and future research.	Line 243-333
**OTHER INFORMATION**			
Registration and protocol	24a	Provide registration information for the review, including register name and registration number, or state that the review was not registered.	-
	24b	Indicate where the review protocol can be accessed, or state that a protocol was not prepared.	-
	24c	Describe and explain any amendments to information provided at registration or in the protocol.	-
Support	25	Describe sources of financial or non-financial support for the review, and the role of the funders or sponsors in the review.	Title page – Funding
Competing interests	26	Declare any competing interests of review authors.	Title page – Disclosures
Availability of data, code and other materials	27	Report which of the following are publicly available and where they can be found: template data collection forms; data extracted from included studies; data used for all analyses; analytic code; any other materials used in the review.	Data availability statement

Supplemental Table 2. Detailed search strategy

Database	Search strategy
**PubMed (MEDLINE)**	("Cardiac Resynchronization Therapy"[Mesh] OR "cardiac resynchronization therapy"[Title/Abstract] OR "cardiac resynchronization"[Title/Abstract] OR CRT[Title/Abstract] OR "biventricular pacing"[Title/Abstract] OR "cardiac resynchronisation"[Title/Abstract]) AND ("cardiac magnetic resonance"[Title/Abstract] OR "cardiovascular magnetic resonance"[Title/Abstract] OR CMR[Title/Abstract] OR "late gadolinium enhancement"[Title/Abstract] OR LGE[Title/Abstract] OR "myocardial fibrosis"[Title/Abstract] OR "myocardial scar"[Title/Abstract])
**Web of Science**	TS=(("cardiac resynchronization therapy" OR "cardiac resynchronization" OR CRT OR "biventricular pacing" OR "cardiac resynchronisation") AND ("cardiac magnetic resonance" OR "cardiovascular magnetic resonance" OR CMR OR "late gadolinium enhancement" OR LGE OR "myocardial fibrosis" OR "myocardial scar"))
**Scopus**	TITLE-ABS(("cardiac resynchronization therapy" OR "cardiac resynchronization" OR CRT OR "biventricular pacing" OR "cardiac resynchronisation") AND ("cardiac magnetic resonance" OR "cardiovascular magnetic resonance" OR CMR OR "late gadolinium enhancement" OR LGE OR "myocardial fibrosis" OR "myocardial scar"))

Supplemental Table 3. List of full texts excluded and reasons for exclusion

**First Author, Year**	**Reason for exclusion**
**Ypenburg, 2007** [1]	Population not fulfilling inclusion criteria in this meta-analysis
**Chalil, 2007** [2]	
**Storkås, 2020** [3]	
**El-Damaty, 2024** [4]	
**Jansen, 2008** [5]	No specific data of NICM population available
**Taylor, 2010** [6]	
**Cochet, 2013** [7]	
**Sohal, 2014**[8]	
**Sohal, 2014** [9]	
**Ahmed, 2016** [10]	
**Taylor, 2016** [11]	
**Gage, 2018** [12]	
**Nguyên, 2018** [13]	
**Harb, 2019** [14]	
**Bilchick, 2020** [15]	
**Aalen, 2020** [16]	
**Sidhu, 2021** [17]	
**Gao, 2021** [18]	
**Bivona, 2022** [19]	
**Rathi, 2022** [20]	
**White, 2006** [21]	No specific evaluation of the outcomes defined in the meta-analysis
**Bilchick, 2008** [22]	
**Loring, 2013** [23]	
**Hopman, 2023** [24]	
**Marsan, 2009** [25]	No data about outcomes stratified according to LGE presence or absence
**Yokokawa, 2009** [26]	
**Petryka, 2012** [27]	
**Ellims, 2013** [28]	
**Chen, 2016** [29]	
**Werys, 2016** [30]	
**Höke, 2017** [31]	
**Shlevkov, 2018** [32]	
**Stukalova, 2019** [33]	
**Chen, 2024** [34]	
**Leyva, 2012** [35]	A more recent series is included
**Leyva, 2017** [36]	
**Acosta, 2018** [37]	
**Degtiarova, 2021** [38]	
**Chen, 2023** [39]	
**Larsen, 2023** [40]	

Abbreviations: LGE = late gadolinium enhancement; NICM = non-ischemic cardiomyopathy.

Supplemental Table 4. Newcastle–Ottawa Scale (NOS) Quality Assessment of Included Studies Evaluating Echocardiographic Response to CRT

First Author, Year	Selection				Comparability	Outcome			Total Score
	S1	S2	S3	S4	C	O1	O2	O3	
**Fernández-Armenta, 2012** [41]	1	1	1	1	0	1	1	1	7/9
**Andre, 2018** [42]	1	1	1	1	0	1	1	1	7/9
**Linhart, 2020** [43]	1	1	1	1	0	1	1	1	7/9
**Duchenne, 2023** [44]	1	1	1	1	0	1	1	1†	7/9
**Maffessanti, 2023** [45]	1	1	1	1	0	1	1	1	7/9
**Ponnusamy, 2023** [46]	1	1	1	1	0	1	1	1	7/9
**Song, 2023** [47]	1	1	1	1	1	1	1	1	8/9
**Ma, 2024** [48]	1	1	1	1	0	1	1	1	7/9
**Anguera, 2025** [49]	1	1	1	1	1	1	1	1	8/9

†Patients without cardiac MRI were excluded prior to follow-up and were not considered as loss to follow-up for NOS scoring.

Abbreviations: NOS = Newcastle–Ottawa Scale; S1 = representativeness of exposed cohort; S2 = selection of non-exposed cohort; S3 = ascertainment of exposure; S4 = outcome absent at start; C = comparability; O1 = assessment of outcome; O2 = follow-up long enough; O3 = adequacy of follow-up.

Supplemental Table 5. Newcastle–Ottawa Scale (NOS) Quality Assessment of Included Studies Evaluating Ventricular Arrhythmic Events

First Author, Year	Selection				Comparability	Outcome			Total Score
	S1	S2	S3	S4	C	O1	O2	O3	
**Fernández-Armenta, 2012** [41]	1	1	1	1	0	1	1	1	7/9
**Andre, 2018** [42]	1	1	1	1	0	1	1	1	7/9
**Linhart, 2020** [43]	1	1	1	1	0	1	1	1	7/9
**Leyva, 2022** [50]	1	1	1	1	0	1	1	1	7/9
**Ponnusamy, 2023** [46]	1	1	1	1	0	0	1	1	6/9
**Anguera, 2025** [49]	1	1	1	1	1	1	1	1	8/9

Abbreviations: NOS = Newcastle–Ottawa Scale; S1 = representativeness of exposed cohort; S2 = selection of non-exposed cohort; S3 = ascertainment of exposure; S4 = outcome absent at start; C = comparability; O1 = assessment of outcome; O2 = follow-up long enough; O3 = adequacy of follow-up.

Supplemental Table 6. Criteria to Define Nonischemic Cardiomyopathy in Each Study

First Author, Year	Definition of NICM
**Fernández-Armenta, 20** [41]	Absence of significant CAD (>70% stenosis of a coronary artery on angiography), history of MI, or evidence of ischemia on image stress testing.
**Andre, 2018** [42]	Absence of significant CAD (not further specified). Exclusion criteria: previously implanted PM.
**Linhart, 2020** [43]	Absence of significant CAD (>75% of two or more epicardial vessels, or left main or proximal left anterior descending artery stenosis >75%) or history of MI
**Leyva, 2022** [50]	Diagnosis of NICM was made if the following criteria were met: 1) no history of MI, PCI, or coronary artery bypass; 2) absence of significant CAD (coronary angiography showing >70% stenosis in 1 or more coronary artery or >50% stenosis in the left main stem); and 3) absence of congenital heart disease, primary valvular disease, infiltrative cardiomyopathy, and channelopathies. Exclusion criteria: HCM, ARVC, and infiltrative heart disease; implanted CIEDs
**Duchenne, 2023** [44]	Not specified. Exclusion criteria: severe AS.
**Maffessanti, 2023** [45]	Exclusion criteria: CAD requiring revascularization; implantation of PM; severe AS; HIV.
**Ponnusamy, 2023** [46]	Absence of significant CAD (not specified) or history of MI, and absence of other identifiable causes for cardiomyopathy.
**Song, 2023** [47]	Exclusion criteria: ischemic heart disease, defined as previous MI or significant CAD (>50% stenosis in a major coronary artery), infiltrative disease, or valvular cardiomyopathy.
**Ma, 2024** [48]	Not specified.
**Anguera, 2025** [49]	Exclusion criteria: Absence of significant CAD (>70 % luminal stenosis in a major coronary artery or > 50 % in the left main coronary artery), history of MI or subendocardial LGE, primary severe valve abnormalities, or diagnosis of other cardiomyopathies such as HCM, ARVC, cardiac amyloidosis or congenital heart disease.
Abbreviations: ARVC = arrhythmogenic right ventricular cardiomyopathy; AS = aortic stenosis; CAD = coronary artery disease; CIEDs = cardiac implantable electronic devices; HCM = hypertrophic cardiomyopathy; HIV = human immunodeficiency virus; LGE = late gadolinium enhancement; MI = myocardial infarction; NICM = nonischemic cardiomyopathy; PCI = percutaneous coronary intervention; PM = pacemaker.	

Supplemental Table 7. Methods for Evaluation of Late Gadolinium Enhancement in Included Studies

First Author, Year	Method for Evaluation of LGE
**Fernández-Armenta, 2012**[41]	Intensity >2 SDs of normal myocardium
**Andre, 2018** [42]	Visual analysis
**Linhart, 2020** [43]	Visual analysis
**Leyva, 2022** [50]	Visual analysis
**Duchenne, 2023** [44]	Automated quantification using a FWHM-based algorithm [51]
**Maffessanti, 2023** [45]	FWHM method [52]
**Ponnusamy, 2023** [46]	Visual analysis
**Song, 2023** [47]	Visual analysis
**Ma, 2024** [48]	Visual analysis
**Anguera, 2025** [49]	Visual analysis
Abbreviations: LGE = late gadolinium enhancement; FWHM = full width at half maximum; SD = standard deviation.	

Supplemental Table 8. Odds Ratio for CRT Response Using Fixed- and Random-Effects Models

Model	Pooled OR (95% CI), p value	Heterogeneity (Q test p value)	I^2 (%)	Tau^2
Fixed-effect (Inverse-Variance)	3.520 (2.539-4.881), <0.001	0.191	28.49	-
Random-effects (REML)	3.520 (2.539-4.881), <0.001	0.191	0.00	0.000

The fixed-effect model was estimated using the inverse-variance method, and the random-effects model was estimated using restricted maximum likelihood (REML).

Abbreviations: CI = confidence interval; OR = odds ratio; REML: restricted maximum likelihood

Supplemental Table 9. Analysis of Publication Bias

		p value
**CRT Response**	Egger's Test	0.582
	Peters' Test	0.661
**Arrhythmic Events**	Egger's Test	0.539
	Peters' Test	0.926

Results of Egger's and Peters' tests for small-study effects for both clinical endpoints.

Supplemental Table 10. Odds Ratio for Ventricular Arrhythmias Using Fixed- and Random-Effects Models

Model	Pooled OR (95% CI), p value	Heterogeneity (Q test p value)	I^2 (%)	Tau^2
Fixed-effect (Inverse-Variance)	0.061 (0.024-0.153), <0.001	0.656	0.00	-
Random-effects (REML)	0.061 (0.024-0.153), <0.001	0.656	0.00	0.000

The fixed-effect model was estimated using the inverse-variance method, and the random-effects model was estimated using restricted maximum likelihood (REML).

Abbreviations: CI = confidence interval; OR = odds ratio; REML: restricted maximum likelihood

Supplemental Table 11. Subgroup and Sensitivity Analyses of the Association Between LGE and Echocardiographic Response to CRT

Analysis	Subgroup/ restriction	CRT Response OR (95% CI)	p value for pooled effect	P for subgroup difference	Patients (n)	Studies (n)
**Subgroup analyses**						
**Study design**	**Prospective** [41–46]	2.71 (1.68-4.35)	<0.001	0.136	425	6
	**Retrospective** [47–49]	4.51 (2.80-7.26)	<0.001		406	3
**LGE assesment**	**Visual** [42,43,46–49]	3.69 (2.60-5.24)	<0.001	0.645	692	6
	**Quantitative/semi-quantitative**[a] [41,44,45]	2.68 (0.72-9.95)	0.140		138	3
**Response definition**	**LVEF improvement** [46–49]	4.40 (2.92-6.63)	<0.001	0.077	518	4
	**LVESV reduction** [41–45]	2.38 (1.39-4.10)	0.002		313	5
**Sensitivity analysis**						
	**Excluding studies with small sample size** [43,44,46–49]	3.88 (2.76-5.45)	<0.001	-	747	6
	**Studies assessing both endpoints (CRT response and ventricular arrhythmias)**[41–43,46,49]	2.89 (1.87-4.29)	<0.001	-	489	5

ORs were pooled using random-effects models. Subgroup differences were assessed using the between-group Q test, whereas sensitivity analyses repeated the meta-analysis in restricted study sets.
[a]Quantitative/semi-quantitative assessment refers to studies using signal intensity thresholds (≥2 SDs above normal myocardium), automated quantification, or full-width half-maximum (FWHM) methods .
Abbreviations: CI = confidence interval; CRT = cardiac resynchronization therapy; FWHM = full-width at half maximum; LGE = late gadolinium enhancement; LVEF = left ventricular ejection fraction; LVESV = left ventricular end-systolic volume; OR = odds ratio.

Supplemental Table 12. Subgroup and Sensitivity Analyses of the Association Between LGE and Ventricular Arrhythmic Events

Analysis	Subgroup/ restriction	Arrhythmic Events OR (95% CI)	p value for pooled effect	P for subgroup difference	Patients (n)	Studies (n)
**Subgroup analyses**						
**Study design**	**Prospective** [41–43,46,50]	0.07 (0.03-0.19)	<0.001	0.338	578	5
	**Retrospective** [49]	0.02 (0.00-0.28)	0.004		167	1
**LGE assesment**	**Visual** [42,43,46,49,50]	0.07 (0.03-0.18)	<0.001	0.538	708	5
	**Quantitative/semi-quantitative**[a] [41]	0.03 (0.00-0.49)	0.015		37	1
**Sensitivity analysis**						
	**Arrhythmic endpoint including appropriate ICD therapies** [41,43,46,49,50]	0.06 (0.02-0.14)	<0.001	-	709	5
	**Excluding studies with small sample size** [43,46,49,50]	0.06 (0.02-0.16)	<0.001	-	672	4
	**Studies assessing both endpoints (CRT response and ventricular arrhythmias)** [41–43,46,49]	0.04 (0.01-0.13)	<0.001	-	490	5

ORs were pooled using random-effects models. Subgroup differences were assessed using the between-group Q test, whereas sensitivity analyses repeated the meta-analysis in restricted study sets.

[a]Quantitative/semi-quantitative assessment refers to studies using signal intensity thresholds (≥2 SDs above normal myocardium), automated quantification, or full-width half-maximum (FWHM) methods.

Abbreviations: CI = confidence interval; CRT = cardiac resynchronization therapy; FWHM = full-width at half maximum; ICD = implantable cardioverter-defibrillator; LGE = late gadolinium enhancement; OR = odds ratio.

SUPPLEMENTAL FIGURES

Supplemental Figure 1. Funnel Plot of CRT Response

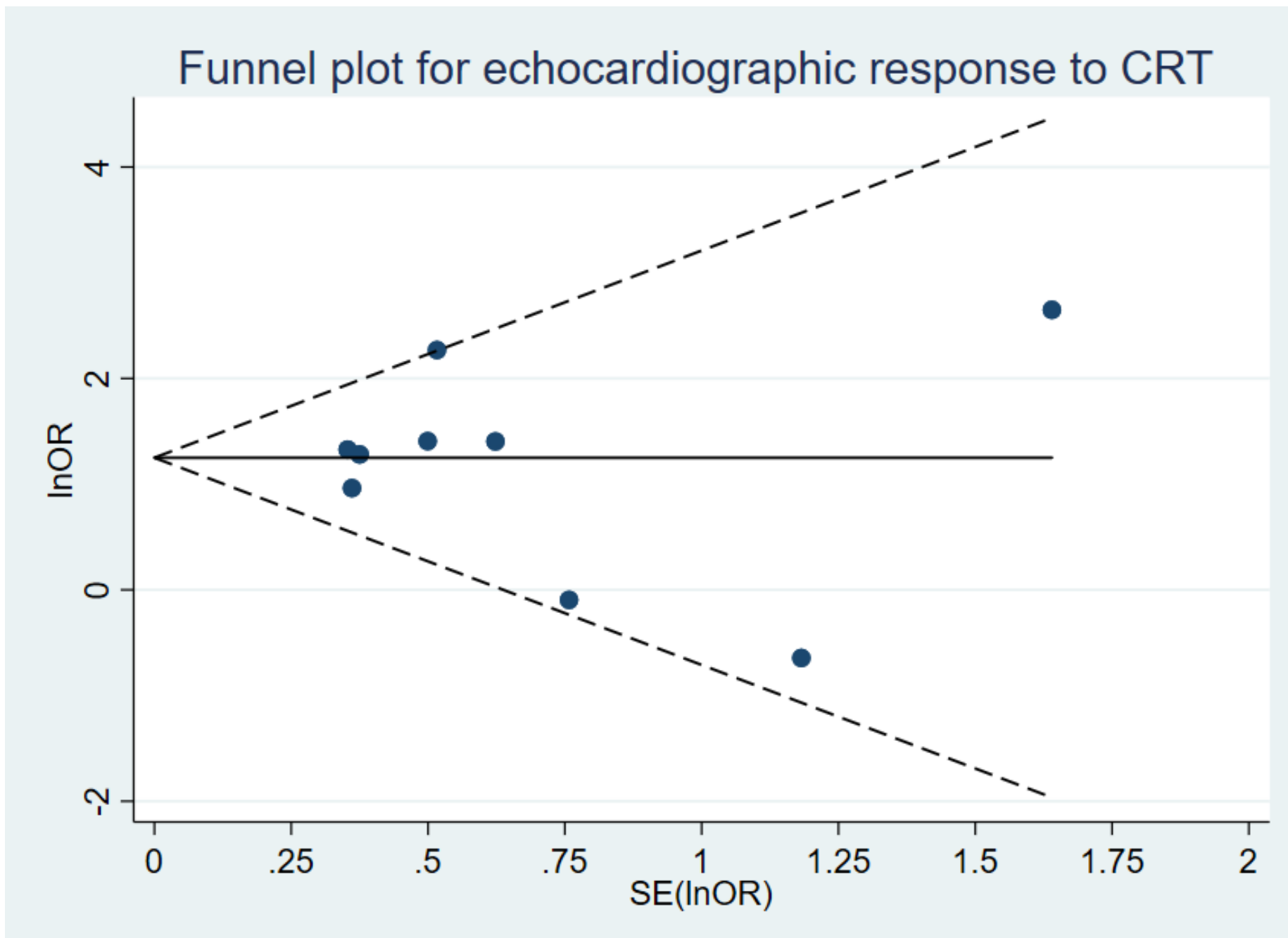

Funnel plot evaluating potential publication bias for echocardiographic response to CRT. Circles represent individual studies, plotted by effect size (lnOR) and precision (SE[lnOR]). The solid line indicates the pooled effect estimate, and dashed lines the pseudo–95% confidence limits.

Abbreviations: CRT = cardiac resynchronization therapy; lnOR = natural logarithm of the odds ratio; SE = standard error.

Supplemental Figure 2. Funnel Plot of Ventricular Arrhythmic Events

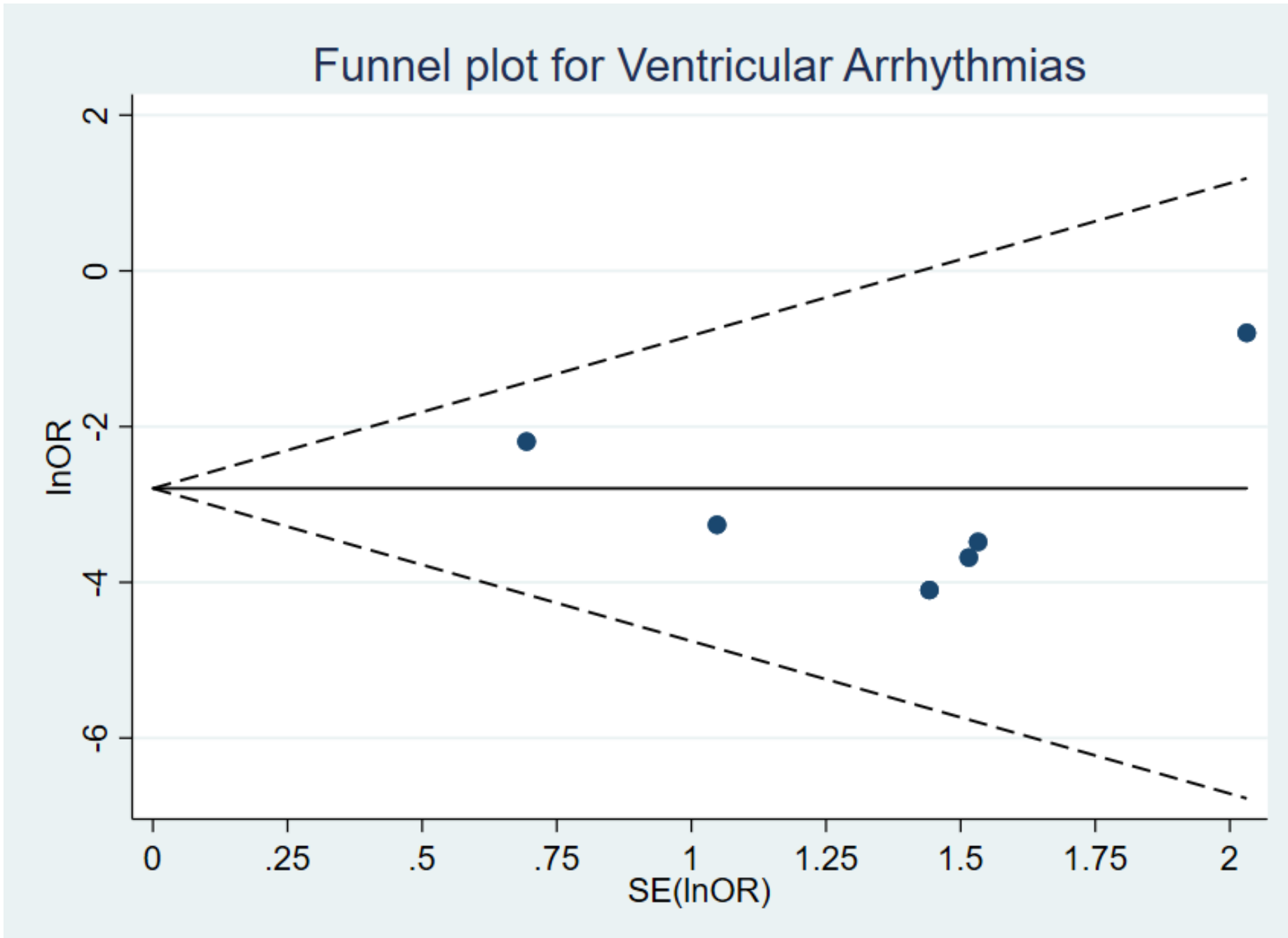

Funnel plot evaluating potential publication bias for the arrhythmic endpoint. Circles represent individual studies plotted by effect size (lnOR) and precision (SE[lnOR]). The solid line indicates the pooled effect estimate, and dashed lines the pseudo–95% confidence limits.

Abbreviations: lnOR = natural logarithm of odds ratio; SE = standard error.

SUPPLEMENTAL REFERENCES

1. Ypenburg C, Roes SD, Bleeker GB, et al. Effect of total scar burden on contrast-enhanced magnetic resonance imaging on response to cardiac resynchronization therapy. *Am J Cardiol*. 2007;99(5):657-660.
2. Chalil S, Stegemann B, Muhyaldeen SA, et al. Effect of posterolateral left ventricular scar on mortality and morbidity following cardiac resynchronization therapy. *Pacing Clin Electrophysiol*. 2007;30(10):1201-1209.
3. Storkås HS, Hansen TF, Tahri JB, et al. Left axis deviation in patients with left bundle branch block is a marker of myocardial disease associated with poor response to cardiac resynchronization therapy. *J Electrocardiol*. 2020;63:147-152.
4. El-Damaty A, Sayed M, El-Maghawry M, Kandil H, Hassan M. Utility of Cardiac Magnetic Resonance in Assessing Arrhythmic Risk in Patients With Nonischemic Cardiomyopathy Undergoing Biventricular Pacing. *Pacing Clin Electrophysiol*. 2024;47(11):1528-1538.
5. Jansen AH, Bracke F, van Dantzig JM, et al. The influence of myocardial scar and dyssynchrony on reverse remodeling in cardiac resynchronization therapy. *Eur J Echocardiogr*. 2008;9(4):483-488.
6. Taylor AJ, Elsik M, Broughton A, et al. Combined dyssynchrony and scar imaging with cardiac magnetic resonance imaging predicts clinical response and long-term prognosis following cardiac resynchronization therapy. *Europace*. 2010;12(5):708-713.
7. Cochet H, Denis A, Ploux S, et al. Pre- and intra-procedural predictors of reverse remodeling after cardiac resynchronization therapy: An MRI study. *J Cardiovasc Electrophysiol*. 2013;24(6):682-691.

8. Sohal M, Amraoui S, Chen Z, et al. Combined identification of septal flash and absence of myocardial scar by cardiac magnetic resonance imaging improves prediction of response to cardiac resynchronization therapy. *J Interv Card Electrophysiol*. 2014;40(2):179-190.

9. Sohal M, Duckett SG, Zhuang X, et al. A prospective evaluation of cardiovascular magnetic resonance measures of dyssynchrony in the prediction of response to cardiac resynchronization therapy. *J Cardiovasc Magn Reson*. 2014;16(1):58

10. Ahmed W, Samy W, Tayeh O, Behairy N, Abd El Fattah A. Left ventricular scar impact on left ventricular synchronization parameters and outcomes of cardiac resynchronization therapy. *Int J Cardiol*. 2016;222:665-670.

11. Taylor RJ, Umar F, Panting JR, Stegemann B, Leyva F. Left ventricular lead position, mechanical activation, and myocardial scar in relation to left ventricular reverse remodeling and clinical outcomes after cardiac resynchronization therapy: A feature-tracking and contrast-enhanced cardiovascular magnetic resonance study. *Heart Rhythm*. 2016;13(2):481-489.

12. Gage RM, Khan AH, Syed IS, et al. Twelve-Lead ECG Optimization of Cardiac Resynchronization Therapy in Patients With and Without Delayed Enhancement on Cardiac Magnetic Resonance Imaging. *J Am Heart Assoc*. 2018;7(23):e009559.

13. Nguyên UC, Claridge S, Vernooy K, et al. Relationship between vectorcardiographic QRS_{area}, myocardial scar quantification, and response to cardiac resynchronization therapy. *J Electrocardiol*. 2018;51(3):457-463.

14. Harb SC, Toro S, Bullen JA, et al. Scar burden is an independent and incremental predictor of cardiac resynchronisation therapy response. *Open Heart*. 2019;6(2):e001067.

15. Bilchick KC, Auger DA, Abdishektaei M, et al. CMR DENSE and the Seattle Heart Failure Model Inform Survival and Arrhythmia Risk After CRT. *JACC Cardiovasc Imaging*. 2020;13(4):924-936.

16. Aalen JM, Donal E, Larsen CK, et al. Imaging predictors of response to cardiac resynchronization therapy: left ventricular work asymmetry by echocardiography and septal viability by cardiac magnetic resonance. *Eur Heart J*. 2020;41(39):3813-3823.

17. Sidhu BS, Gould J, Elliott MK, et al. Clinical effectiveness of a dedicated cardiac resynchronization therapy pre-assessment clinic incorporating cardiac magnetic resonance imaging and cardiopulmonary exercise testing on patient selection and outcomes. *Int J Cardiol Heart Vasc*. 2021;34:100800.

18. Gao X, Abdi M, Auger DA, et al. Cardiac Magnetic Resonance Assessment of Response to Cardiac Resynchronization Therapy and Programming Strategies. *JACC Cardiovasc Imaging*. 2021;14(12):2369-2383.

19. Bivona DJ, Tallavajhala S, Abdi M, et al. Cardiac magnetic resonance defines mechanisms of sex-based differences in outcomes following cardiac resynchronization therapy. *Front Cardiovasc Med*. 2022;9:1007806.

20. Rathi A, Kanee L, Limoges M, et al. Relationship Between Left Ventricular Strain Assessment by Cardiac Magnetic Resonance Imaging and Response to Cardiac Resynchronization Therapy. *J Thorac Imaging*. 2022;37(4):W58-W59.

21. White JA, Yee R, Yuan X, et al. Delayed Enhancement Magnetic Resonance Imaging Predicts Response to Cardiac Resynchronization Therapy in Patients With Intraventricular Dyssynchrony. *J Am Coll Cardiol*. 2006;48(10):1953-1960.

22. Bilchick KC, Dimaano V, Wu KC, et al. Cardiac Magnetic Resonance Assessment of Dyssynchrony and Myocardial Scar Predicts Function Class Improvement Following Cardiac Resynchronization Therapy. *JACC Cardiovasc Imaging*. 2008;1(5):561-568.

23. Loring Z, Strauss DG, Gerstenblith G, Tomaselli GF, Weiss RG, Wu KC. Cardiac MRI scar patterns differ by sex in an implantable cardioverter-defibrillator and cardiac resynchronization therapy cohort. *Heart Rhythm*. 2013;10(5):659-665.

24. Hopman LHGA, van der Lingen ACJ, van Pouderoijen N, et al. Cardiac Magnetic Resonance Imaging-Derived Left Atrial Characteristics in Relation to Atrial Fibrillation Detection in Patients With an Implantable Cardioverter-Defibrillator. *J Am Heart Assoc*. 2023;12(15):e028014.

25. Marsan NA, Westenberg JJM, Ypenburg C, et al. Magnetic resonance imaging and response to cardiac resynchronization therapy: Relative merits of left ventricular dyssynchrony and scar tissue. *Eur Heart J*. 2009;30(19):2360-2367.

26. Yokokawa M, Tada H, Toyama T, et al. Magnetic resonance imaging is superior to cardiac scintigraphy to identify nonresponders to cardiac resynchronization therapy. *Pacing Clin Electrophysiol*. 2009;32 Suppl 1:S57-S62. 27.

27. Petryka J, Miśko J, Przybylski A, et al. Magnetic resonance imaging assessment of intraventricular dyssynchrony and delayed enhancement as predictors of response to cardiac resynchronization therapy in patients with heart failure of ischaemic and non-ischaemic etiologies. *Eur J Radiol*. 2012;81(10):2639-2647.

28. Ellims AH, Pfluger H, Elsik M, Butler MJ, Hare JL, Taylor AJ. Utility of cardiac magnetic resonance imaging, echocardiography and electrocardiography for the prediction of clinical

response and long-term survival following cardiac resynchronisation therapy. *Int J Cardiovasc Imaging*. 2013;29(6):1303-1311.

29. Chen Z, Sohal M, Sammut E, et al. Focal but Not Diffuse Myocardial Fibrosis Burden Quantification Using Cardiac Magnetic Resonance Imaging Predicts Left Ventricular Reverse Modeling Following Cardiac Resynchronization Therapy. *J Cardiovasc Electrophysiol*. 2016;27(2):203-209.

30. Werys K, Petryka-Mazurkiewicz J, Błaszczyk Ł, et al. Cine dyscontractility index: A novel marker of mechanical dyssynchrony that predicts response to cardiac resynchronization therapy. *J Magn Reson Imaging*. 2016;44(6):1483-1492.

31. Höke U, Khidir MJ, van der Geest RJ, et al. Relation of Myocardial Contrast-Enhanced T_1 Mapping by Cardiac Magnetic Resonance to Left Ventricular Reverse Remodeling After Cardiac Resynchronization Therapy in Patients With Nonischemic Cardiomyopathy. *Am J Cardiol*. 2017;119(9):1456-1462.

32. Shlevkov NB, Zhambeev AA, Gasparyan AZ, Shitov VN, Stukalova OV. Characteristic of fibrotic myocardial lesions associated with life-threatening ventricular tachyarrhythmias in patients with ischemic and non-ischemic cardiomyopathies. *Ter Arkh*. 2018;90(9):42-47.

33. Stukalova OV, Mironova NA, Utsumueva MD, et al. The effectiveness of cardiac resynchronization therapy in patients with chronic heart failure of various origin depending on the structural myocardial injury in cardiac magnetic resonance imaging. *Russ J Cardiol*. 2019;24(12):22-32.

34. Chen Z, Ma X, Wu S, et al. A Comparison of the Association of Septal Scar Burden on Responses to LBBAP-CRT and BVP-CRT. *JACC Clin Electrophysiol*. 2024;10(7 Pt 1):1439-1451.

35. Leyva F, Taylor RJ, Foley PWX, et al. Left ventricular midwall fibrosis as a predictor of mortality and morbidity after cardiac resynchronization therapy in patients with nonischemic cardiomyopathy. *J Am Coll Cardiol*. 2012;60(17):1659-1667.

36. Leyva F, Zegard A, Acquaye E, et al. Outcomes of Cardiac Resynchronization Therapy With or Without Defibrillation in Patients With Nonischemic Cardiomyopathy. *J Am Coll Cardiol*. 2017;70(10):1216-1227.

37. Acosta J, Fernández-Armenta J, Borràs R, et al. Scar Characterization to Predict Life-Threatening Arrhythmic Events and Sudden Cardiac Death in Patients With Cardiac Resynchronization Therapy: The GAUDI-CRT Study. *JACC Cardiovasc Imaging*. 2018;11(4):561-572.

38. Degtiarova G, Claus P, Duchenne J, et al. Left ventricular regional glucose metabolism in combination with septal scar extent identifies CRT responders. *Eur J Nucl Med Mol Imaging*. 2021;48(8):2437-2446.

39. Chen Z, Ma X, Gao Y, et al. Cardiac magnetic resonance-derived myocardial scar is associated with echocardiographic response and clinical prognosis of left bundle branch area pacing for cardiac resynchronization therapy. *Europace*. 2023;25(11):euad326.

40. Larsen CK, Smiseth OA, Duchenne J, et al. Cardiac Magnetic Resonance Identifies Responders to Cardiac Resynchronization Therapy with an Assessment of Septal Scar and Left Ventricular Dyssynchrony. *J Clin Med*. 2023;12(22):7182.

41. Fernández-Armenta J, Berruezo A, Mont L, et al. Use of myocardial scar characterization to predict ventricular arrhythmia in cardiac resynchronization therapy. *Europace*. 2012;14(11):1578-1586.

42. Andre C, Piver E, Perault R, et al. Galectin-3 predicts response and outcomes after cardiac resynchronization therapy. *J Transl Med*. 2018;16(1):299.

43. Linhart M, Doltra A, Acosta J, et al. Ventricular arrhythmia risk is associated with myocardial scar but not with response to cardiac resynchronization therapy. *Europace*. 2020;22(9):1391-1400.

44. Duchenne J, Larsen CK, Cvijic M, et al. Mechanical Dyssynchrony Combined with Septal Scarring Reliably Identifies Responders to Cardiac Resynchronization Therapy. *J Clin Med*. 2023;12(18):6108.

45. Maffessanti F, Jadczyk T, Wilczek J, et al. Electromechanical factors associated with favourable outcome in cardiac resynchronization therapy. *Europace*. 2023;25(2):546-553.

46. Ponnusamy SS, Ganesan V, Ramalingam V, et al. MAgnetic resonance imaging based DUal lead cardiac Resynchronization therapy: A prospectIve Left Bundle Branch Pacing study (MADURAI LBBP study). *Heart Rhythm*. 2023;20(8):1119-1127.

47. Song Y, Chen X, Yang K, et al. Cardiac MRI-derived Myocardial Fibrosis and Ventricular Dyssynchrony Predict Response to Cardiac Resynchronization Therapy in Patients with Nonischemic Dilated Cardiomyopathy. *Radiol Cardiothorac Imaging*. 2023;5(5):e220127.

48. Ma X, Chen Z, Song Y, et al. CMR feature tracking–based left atrial mechanics predicts response to cardiac resynchronization therapy and adverse outcomes. *Heart Rhythm*. 2024;21(8):1354-1362.

49. Anguera I, Faga V, Jimenez-Candil J, et al. Late gadolinium enhancement and outcome of cardiac resynchronization therapy in non-ischemic cardiomyopathy. *Int J Cardiol*. 2025;418:132618.

50. Leyva F, Zegard A, Okafor O, et al. Myocardial Fibrosis Predicts Ventricular Arrhythmias and Sudden Death After Cardiac Electronic Device Implantation. *J Am Coll Cardiol*. 2022;79(7):665-678.

51. Engblom H, Tufvesson J, Jablonowski R, et al. A new automatic algorithm for quantification of myocardial infarction imaged by late gadolinium enhancement cardiovascular magnetic resonance: experimental validation and comparison to expert delineations in multi-center, multi-vendor patient data. *J Cardiovasc Magn Reson*. 2016;18(1):27.

52. Flett AS, Hasleton J, Cook C, et al. Evaluation of techniques for the quantification of myocardial scar of differing etiology using cardiac magnetic resonance. *JACC Cardiovasc Imaging*. 2011;4(2):150-156.
